# Successful Management of Intractable Hiccups in Diabetic Ketoacidosis: A Case Report on the Efficacy of Inhaled Nebulized Lidocaine

**DOI:** 10.7759/cureus.88287

**Published:** 2025-07-19

**Authors:** UFN Rizwanullah, Abdulgafar Dare Ibrahim, Fardin Akbar Hyderi, Nadira Tamanna Tamim, Vasudha Batta, Shivani Shah

**Affiliations:** 1 Internal Medicine, Hayatabad Medical Complex Peshawar, Peshawar, PAK; 2 Internal Medicine, Mississippi Baptist Medical Center, Jackson, USA; 3 Medicine, The Partners Care, New York, USA; 4 Medicine, The Grand Rehabilitation and Nursing Home, New York, USA; 5 Medicine, Sri Guru Ram Das Institute of Medical Sciences and Research, Amritsar, IND; 6 Medicine, Caribbean Medical University School of Medicine, Chicago, USA

**Keywords:** case report, diabetic ketoacidosis, hiccups management, intractable hiccups, nebulized lidocaine

## Abstract

Intractable hiccups are a rare but distressing symptom that can significantly impair a patient's quality of life, particularly in those with underlying medical conditions such as diabetic ketoacidosis (DKA). This case report describes a 59-year-old male patient with a history of type 2 diabetes mellitus and hypertension who presented to the emergency department with persistent hiccups lasting for five days, coinciding with an exacerbation of his diabetes leading to DKA. Despite initial treatments with metoclopramide, chlorpromazine, and baclofen, the hiccups persisted, causing considerable distress. The patient was subsequently treated with inhaled nebulized lidocaine at a dosage of 150 mg daily for two days. Remarkably, within 24 hours, the patient experienced a significant reduction in hiccup frequency, and by the end of the treatment course, the hiccups had completely resolved. This case highlights the potential efficacy of nebulized lidocaine as an alternative treatment for intractable hiccups associated with DKA, particularly when conventional therapies fail. Given the promising results, further research is warranted to explore the mechanisms and broader applicability of this treatment approach in similar patient populations.

## Introduction

Intractable hiccups are a rare but distressing symptom that can significantly impact a patient’s quality of life, particularly in those with underlying medical conditions such as diabetic ketoacidosis (DKA) [1]. Hiccups are involuntary contractions of the diaphragm followed by a sudden closure of the vocal cords, producing the characteristic "hic" sound. While most cases of hiccups are transient and self-limiting, some individuals experience persistent or intractable hiccups that can last for days or even weeks, leading to complications such as dehydration, malnutrition, and sleep disturbances. The prevalence of intractable hiccups can be particularly notable in patients with DKA, negatively affecting the quality of life in approximately 10% of affected individuals [2].

Management of intractable hiccups often involves a trial of various pharmacological agents, including metoclopramide, baclofen, and chlorpromazine; however, these treatments may not always provide effective relief and can sometimes result in adverse effects [3]. Intractable hiccups can be challenging to treat, but several pharmacological interventions have shown promise. Intravenous lidocaine has been reported as an effective treatment for severe hiccups [4,5]. In one case, two successive infusions of lidocaine successfully terminated hiccups when other treatments failed [5].

Mechanism of lidocaine in hiccups

Lidocaine, primarily known as a local anesthetic, exerts its effects on hiccups through several mechanisms. Firstly, it blocks voltage-gated sodium channels, which reduces neuronal excitability and decreases the likelihood of ectopic discharges that can trigger the hiccup reflex. Additionally, lidocaine has membrane-stabilizing properties that further diminish neuronal excitability, making it effective in managing hiccups that may have a neurogenic origin. By modulating calcium channels, lidocaine can also influence neurotransmitter release, potentially impacting the pathways involved in the hiccup reflex. Overall, these mechanisms contribute to lidocaine's efficacy in alleviating hiccups during medical procedures [5].

In light of this, alternative therapies such as nebulized lidocaine have been explored for their potential efficacy in alleviating persistent hiccups. Notably, a case has been reported where nebulized lidocaine was used effectively to manage intractable hiccups in a patient, demonstrating its potential as a therapeutic option [6]. This innovative approach highlights the importance of addressing refractory symptoms in the context of DKA, ensuring that patients receive holistic care that encompasses both metabolic stabilization and symptomatic relief. Our case report describes a patient with DKA who presented with intractable hiccups and showed a significant response to inhaled nebulized lidocaine, proving to be an effective intervention that significantly improved the patient's condition.

## Case presentation

A 59-year-old male patient with a past medical history of type 2 diabetes mellitus and hypertension presented to the emergency department with persistent hiccups lasting for five days. The hiccups began following an exacerbation of his diabetes, which led to DKA, characterized by a blood glucose level of 450 mg/dL and a pH of 7.29, indicating moderate acidosis (Table 1). He was treated with an insulin drip and fluid resuscitation, which stabilized his metabolic status. Despite this treatment, the hiccups persisted, significantly affecting his ability to eat, sleep, and communicate. Initial management for the hiccups included metoclopramide, baclofen, and chlorpromazine, but these medications failed to provide relief. Due to the refractory nature of the hiccups and the patient's distress, nebulized lidocaine was trialed at a dosage of 150 mg daily for two days. Remarkably, within 24 hours of the first dose, the patient reported a significant reduction in hiccup frequency, and by the end of the treatment course, his hiccups had completely resolved. This case underscores the potential of inhaled lidocaine as an effective and alternative treatment for intractable hiccups associated with DKA, especially when conventional therapies fail.

**Table 1:** Laboratory investigations at presentation

Test	Normal Range	Result
White Blood Cell Count (×10³/µL)	4.0-11.0	14.0
Hemoglobin Level (mg/dL)	11.5-17.5	12.3
Platelet Count (×10³/µL)	150-450	430
Serum Calcium Level (mg/dL)	8.0-10.1	9.2
Blood Urea Nitrogen (mg/dL)	18-46	54
Creatinine Level (mg/dL)	0.42-1.07	1.1
Sodium Concentration (mmol/L)	135-155	138
Potassium Concentration (mmol/L)	3.5-5.2	3.6
Chloride Level (mmol/L)	96-113	111
Random Glucose Level (mg/dL)	70-130	450
Total Bilirubin Level (mg/dL)	0.1-1.1	0.5
Alanine Aminotransferase (IU/L)	10-51	40
Alkaline Phosphatase (IU/L)	35-105	76
C-Reactive Protein (mg/dL)	<0.6	2.4
Blood pH	7.35-7.45	7.29
Bicarbonate Level (mmol/L)	23-28	11

## Discussion

Intractable hiccups can pose a significant challenge in clinical practice, particularly in patients with underlying conditions such as DKA. The pathophysiology of hiccups involves irritation of the phrenic nerve or the diaphragm, which can be triggered by various factors, including metabolic disturbances, gastrointestinal issues, and central nervous system irritants [6]. In the case presented, the patient's hiccups were likely exacerbated by the metabolic derangements associated with DKA. This highlights the need for clinicians to consider hiccups as a potential complication in patients with severe metabolic disorders, as they can lead to considerable distress and impact overall treatment outcomes [7].

Traditional management strategies for intractable hiccups (Table 2), such as the use of metoclopramide, baclofen, and chlorpromazine, may not always yield satisfactory results and can result in adverse effects, as seen in our patient. The failure of these conventional therapies emphasizes the necessity for alternative treatment options. Inhaled nebulized lidocaine has emerged as a promising alternative, with studies suggesting its efficacy in alleviating persistent hiccups [6]. Lidocaine's local anesthetic properties may help mitigate the irritative pathways involved in hiccup reflexes, providing a targeted approach to symptom relief.

**Table 2:** Treatment options for hiccups [7]. IV: Intravenous.

Treatment	Mechanism of Action	Dosage	Notes
Lidocaine	Sodium channel blockade; membrane stabilization	1 mg/kg IV or nebulized	Effective for acute hiccups; may reduce neuronal excitability.
Ephedrine	Central stimulant; may affect diaphragm contractions	5 mg IV	More effective than lidocaine in some studies; rapid onset.
Metoclopramide	Dopamine receptor antagonist; increases gastrointestinal (GI) motility	10 mg IV or orally	Effective for chronic hiccups; also used for nausea.
Baclofen	GABA-B receptor agonist; reduces excitability	5-10 mg orally	Useful for chronic cases; may cause sedation.
Gabapentin	Modulates neurotransmitter release; reduces excitability	300-600 mg orally	Effective for chronic hiccups; well-tolerated.
Chlorpromazine	Dopamine antagonist; affects central nervous system	25-50 mg orally	Can be effective but may have significant side effects.
Acupuncture	Non-pharmacological; stimulates specific points	Varies	Some studies show effectiveness; less conventional.
Dexmedetomidine	Alpha-2 adrenergic agonist; sedative effect	0.5-1 µg/kg IV	Emerging evidence for intraoperative hiccups.

Moreover, nebulized lidocaine has also been used successfully to treat refractory cough, suggesting potential applications for hiccup management [8]. This dual application underscores the versatility of lidocaine in treating both hiccups and cough, making it a valuable therapeutic option in various clinical scenarios [9]. Our case demonstrates the rapid and significant response to nebulized lidocaine, suggesting that it could be a valuable addition to the therapeutic arsenal for managing intractable hiccups, especially in complex cases like DKA. Additionally, pregabalin, a structural analog of GABA that modulates neurotransmitter release, has been effective in treating idiopathic intractable hiccups [10].

The successful use of inhaled lidocaine in this patient underscores the importance of individualized treatment plans in managing refractory symptoms. As the medical community continues to explore innovative therapies, it is crucial to conduct further studies to establish optimal dosing regimens and assess the long-term safety and efficacy of inhaled lidocaine for hiccups. Other interventions mentioned include cervical epidural blocks, phrenic nerve blocks, and various medications such as baclofen, clonazepam, and metoclopramide [3].

Additionally, hiccups should not be overlooked in patient care, particularly in the context of metabolic disorders like DKA, where they can significantly impair quality of life. Various treatments have been explored for managing persistent hiccups, and inhaled lidocaine has shown efficacy in treating refractory cases without significant adverse effects [2,9]. Other novel approaches, such as guided diaphragmatic breathing and relaxation techniques, have also demonstrated effectiveness in resolving persistent hiccups refractory to conventional treatments [11]. This diverse array of treatment options highlights the complexity of managing intractable hiccups and the importance of exploring multiple therapeutic avenues, including pharmacological interventions and non-pharmacological techniques, to achieve symptom resolution in challenging cases. Overall, this case adds to the growing body of literature advocating for the consideration of nebulized lidocaine as a viable treatment option for intractable hiccups, particularly in patients with DKA and other underlying conditions.

## Conclusions

This case illustrates the significant impact of intractable hiccups on the quality of life for patients with diabetic ketoacidosis and highlights the potential of inhaled nebulized lidocaine as an effective treatment option when conventional therapies fail. The rapid response observed in this patient suggests that nebulized lidocaine could be a valuable addition to the therapeutic options available for managing refractory hiccups. Given the promising results, further research is warranted to explore the mechanisms and broader applicability of nebulized lidocaine in treating intractable hiccups across different patient populations, particularly those with complex medical conditions like DKA.
